# Relative Fundamental Frequency: Only for Hyperfunctional Voices? A Pilot Study

**DOI:** 10.3390/bioengineering11050475

**Published:** 2024-05-10

**Authors:** Sol Ferrán, Carla Rodríguez-Zanetti, Octavio Garaycochea, David Terrasa, Carlos Prieto-Matos, Beatriz del Río, Maria Pilar Alzuguren, Secundino Fernández

**Affiliations:** 1 Clínica Universidad de Navarra, 31008 Pamplona, Spain; crodriguezzanetti@unav.es (C.R.-Z.); ogaraycoche@unav.es (O.G.); dterrasa@unav.es (D.T.); cprietom@unav.es (C.P.-M.); bdelr@unav.es (B.d.R.); sfgonzalez@unav.es (S.F.); 2 Medical Engineering Laboratory, School of Medicine, Universidad de Navarra, 31008 Pamplona, Spain; palzuguren@unav.es

**Keywords:** muscle tension dysphonia, relative fundamental frequency, vocal hyperfunction, voice acoustics, vocal offset, vocal onset

## Abstract

(1) Background: Assessing phonatory disorders due to laryngeal biomechanical alterations requires aerodynamic analysis, assessing subglottic pressure, transglottic flow, and laryngeal resistance. This study explores whether the acoustic parameter, the relative fundamental frequency (RFF), can be studied using the current acoustic analysis protocol at the University of Navarra’s voice laboratory and its association with pathologies linked to laryngeal biomechanical alterations. (2) Methods: A retrospective cohort study included patients diagnosed with muscular tension dysphonia, organic lesions of the vocal fold, and vocal fold paralysis (VFP) at the Clínica Universidad de Navarra from 2019 to 2021. Each patient underwent endoscopic laryngeal exploration, followed by acoustic study, RFF calculation, and an aerodynamic study. Additionally, a control group was recruited. (3) Results: 79 patients and 22 controls were studied. Two-way ANOVA showed significant effects for groups and cycles in offset and onset cycles. Statistically significant differences were observed in cycle 1 onset among all groups and in cycles 1 and 2 between the control group and non-healthy groups. (4) Conclusions: RFF is a valuable indicator of phonatory biomechanics, distinguishing healthy and pathological voices and different disorders. RFF in onset cycles offers a cost-effective, accurate method for assessing biomechanical disorders without complex aerodynamic analyses. This study describes RFF values in VFP for the first time, revealing differences regardless of aerodynamic patterns.

## 1. Introduction

The examination of voices in patients with dysphonia is primarily supported by four methods: clinical history and perceptual assessment, acoustic analysis, endoscopic examination, and aerodynamic analysis. Endoscopic examination is the gold standard technique in most laryngeal pathological processes. However, vocal pathology may not always be visually evident, even with sophisticated diagnostic instruments, highlighting the importance of complementing it with other methods [1].

Aerodynamic analysis provides valuable information about laryngeal biomechanics, including parameters such as subglottic pressure (sgP), transglottic flow, and laryngeal resistance. Despite its potential, this technique is not routinely employed in clinical practice, partly because of its reliance on a complex array of equipment and systems that are not easily accessible. Conversely, acoustic analysis, which requires progressively less equipment over time with the advent of user-friendly free software, offers numerous parameters for voice characterization. However, none of these parameters possesses specific values for different vocal pathologies.

Consequently, in the past decade, there has been a heightened focus on a parameter derived from acoustic analysis, that appears to provide insights into laryngeal biomechanics: the relative fundamental frequency (RFF).

**Figure 1:**
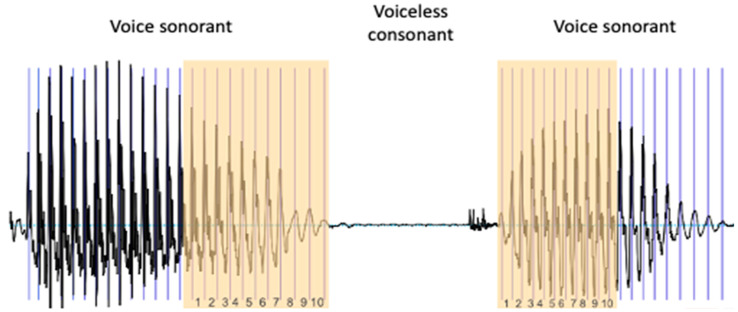
Extraction of the RFF. To calculate the RFF, the last 10 cycles of the vowel preceding the voiceless consonant and the first 10 cycles of the vowel following it are identified.

The RFF is defined as the instantaneous fundamental frequencies ($f_{0}$) of the 10 vocal cycles before and after a voiceless consonant, normalized by the $f_{0}$ of the “steady-state” portions of vocal sounds in a voice–voiceless consonant–voice (VCV) context (Figure 1).

Therefore, the RFF quantifies the amount of short-term $f_{0}$ variation surrounding the production of the voiceless consonant. Instantaneous $f_{0}$ values are normalized to the reference $f_{0}$ values of the cycles furthest from the consonant: “offset cycle” 1 and “onset cycle” 10. These cycles are chosen as reference cycles because they are considered stable parts of the vowels surrounding the consonant in a VCV context. $f_{0}$ normalization enables comparison of RFF values among speakers with different habitual $f_{0}$.

Although the first time this term “Relative Fundamental Frequency” was used was in 2010 [2], the origin of its study comes from many years before, when the difference in $f_{0}$ at the onset of vocalization after a voiceless consonant was observed [3,4]. Hombert et al. [5], after reviewing aerodynamic and vocal fold tension theories that attempted to explain these $f_{0}$ changes at the vocalization onset, found that vertical tension in the larynx was the most viable explanation for the $f_{0}$ perturbations related to vowel consonants. There were already data at that time showing that the positions of the larynx and hyoid bone were lower for voiced consonants than for voiceless consonants [6,7], so their theory was that the vertical position of these structures directly influenced the vocal fold tension and indirectly influenced $f_{0}$.

This, and the physiological mechanisms derived from these and other findings, have led to RFF being postulated as a marker of vocal hyperfunction (VH), a condition characterized by increased levels of laryngeal muscle tension [2].

Muscle tension dysphonia (MTD) is a functional voice disorder characterized by excessive tension of the extrinsic laryngeal musculature during phonation in the absence of an organic or neurological disorder. This increased muscle tension generates an abnormal laryngeal posture during phonation, more superior (the larynx is elevated) and with a certain degree of glottic and/or supraglottic compression [8]. Depending on the mechanism that triggers this increase in muscle tension, MTD can be classified as primary, when this increase in tension is independent of an external mechanism, being an alteration of the phonation mechanism itself; or secondary, when it is a compensatory reaction to a structural alteration of the vocal fold (for example, polyps or nodules) or another mechanism that alters the function or structure of the glottis [9,10].

Another situation where there is a significant alteration in the biomechanical mechanism during phonation is in the injury to the recurrent laryngeal nerve, which provides sensitivity over the glottis and subglottis, and motor innervation over the intrinsic muscles of the larynx, so that its damage makes abduction and adduction of the vocal cord impossible.

The aim of this work was to test whether the RFF could be studied with the acoustic analysis recording protocol used to date in the voice laboratory of the University of Navarra, and whether it distinguished between healthy and pathological voices, on the one hand, and between different pathologies of laryngeal biomechanics, on the other.

## 2. Materials and Methods

### 2.1. Subjects

A retrospective study was carried out including patients attending the voice clinic in the Department of Otolaryngology of the Clínica Universidad de Navarra, between 2019 and 2021, with a diagnosis of muscular tension dysphonia (MTD), organic lesions of the vocal fold (OL)(polyps or nodules) and vocal fold paralysis (VFP).

The inclusion criteria were the following:

- Male or female patients between 18 and 65 years of age.
- They were seen in the office for dysphonia and underwent endoscopic examination, acoustic analysis, and aerodynamic analysis.
- MTD group inclusion was based on the endoscopic examination (glottic or supraglottic compression) and on an sgP calculated in the aerodynamic analysis >90 mmH_2_0. The acoustic analysis was performed on all patients.
- The organic lesion group inclusion was based on endoscopic examination findings, although it was also supported by acoustic and aerodynamic analysis.
- The vocal cord paralysis group inclusion was based on endoscopic examination findings (absence of complete mobility in one vocal cord), although it was also supported by acoustic and aerodynamic analysis.

In addition, a group of control subjects were included in the study. Recruitment was conducted among the hospital’s employees. To be included in the study, they had to be between 18 and 65 years of age and not present any vocal symptoms or have a history of vocal pathology. They underwent an endoscopic examination (with no pathological findings required for inclusion) and an acoustic analysis.

Based on the recording made for the acoustic analysis, the RFF was extracted.

### 2.2. Equipment

For the voice sample collection, a Yeti Pro Studio microphone (BlueMic) was positioned 30 cm from the patient’s lips. The collected analog acoustic signal was digitized using an A/D converter card, SoundScope/16. A sampling rate of 44,000 Hz and a quantization level of 16 bits were employed. Acoustic analysis used the specialized software, SoundScope 1.2, running on an Apple Power Macintosh 9600/233 computer. This program allowed the recording and quantification of frequency and intensity-dependent acoustic parameters, and the generation of spectrographic analysis. The measurements were performed in an isolated and quiet room, but which was not soundproofed.

### 2.3. Extraction of the RFF

Analysis of the acoustic signal to obtain the RFF was performed using Praat software, a free and freely available public program (v. 6.1.26) [11].

As previously mentioned, RFF is defined as the fundamental frequency of the cycles immediately preceding and following the production of a voiceless consonant, normalized in semitone (ST) form by the “steady-state” fundamental frequencies of the vocalization preceding and following the consonant (during a VCV utterance).

These patients were asked to reproduce the phoneme/pateki/, repeated three times, in which we selected/ate/as the VCV sequence.

The process for calculating it is as follows:

First, we identify the last 10 cycles of the first sounding vowel (offset cycles). For this, we identify 11 pulses (Figure 1).

The time difference between each glottal pulse must be calculated to determine the duration of each cycle. From the inverse of the period, we calculate the fundamental frequency. Finally, we calculate the RFF of each cycle (in semitones) using the following equation [12]:

$$\mathrm{RFF}\;=\;39.86\;\times \;\log10(f_{0}/f_{0ref})$$

where $f_{0}$ is the fundamental frequency and $f_{0ref}$ is the fundamental frequency of the reference cycle, which is considered the most stable, that is, the one of the cycle farthest from the voiceless consonant (for the offset cycles, the first one, and for the onset cycles, the tenth one) (Table 1).

The same procedure is then repeated with the first 10 cycles of the second voiced vowel (onset cycles).

It may be the case occasionally that an acoustic signal might display considerable irregularity or exhibit glottalization. It is improbable we can accurately estimate the RFF value from such a signal. The reasons why the RFF may be rejected are the following [13]:

- Glottalization
- Voicing of the voiceless consonant
- Failure to reach a steady state
- RFF value(s) are greater than 10 ST in magnitude
- Glottal fry
- Fewer than 10 cycles

### 2.4. Statistical Analysis

A two-way ANOVA was conducted to assess the effects of the group (control, MTD, organic lesions, and vocal fold paralysis), of the cycle and of the interaction for both offset and onset cycles. A significant interaction would indicate a difference in the RFF between groups on some cycles but not others [2]. Subsequently, a Tukey’s multiple comparison test was performed to evaluate the differences between the four groups. In addition, a Fisher’s test was conducted to assess the differences between the groups for each cycle.

A one-way ANOVA was calculated to assess whether statistically significant differences existed between the RFF offset 10 and onset 1 among patients exhibiting different aerodynamic patterns in the OL and VFP groups.

The control group was also compared with the rest of the groups (sick group) using a two-way ANOVA, followed by a Šídák’s multiple comparisons test to study the differences between the different cycles.

## 3. Results

A total of 79 patients and 22 control subjects were included in the study. The acoustic analysis was performed on all of them, but the usual parameters of this analysis (jitter, shimmer, f0, HNR…) are not analyzed in this study, as they are not of relevance to the purpose of this work.

After the RFF analysis, some of the patients had to be withdrawn from the study because the RFF could not be properly extracted:

- In the MTD group: In four patients, we could not extract the RFF. In the first one, it was not possible to reach a steady state; in the second one, there were fewer than 10 cycles in the offset wave; in the third and fourth ones, the offset wave was irregular and aperiodic, so there were not 10 cycles to measure.
- In the OL group: In two patients, we could not extract the RFF. In one of them, because the offset wave was irregular and aperiodic, there were not 10 cycles to measure; In the other one, because it was not possible to reach a steady state and the onset wave was irregular and aperiodic.
- In the VFP group: In two patients, we could not extract the RFF. In the first one, because there were fewer than 10 cycles in the offset wave; in the second one, because the onset wave was irregular and aperiodic, so there were not 10 cycles to measure.

Finally, the study groups were composed of 24 patients in the MTD group, 28 patients in the OL group, and 27 patients in the VFP group. All the subjects in the control group could be measured properly (22 subjects).

The group of patients with MTD consisted of 24 patients: 16 women (66.67%) and 8 men, with a mean age of 45.35 years (SD 15.41). All of them had elevated sgP (a hyperfunctional aerodynamic pattern), as we consider it as a diagnostic criterion for this pathology. The mean value for the sgP was 115.64 mmH_2_O (SD 27.48). The mean for cycle 10 offset was −1.14 ST; the mean for cycle 1 onset was 1.72 ST.

The OL group comprised 28 patients: 21 females (75%) and 7 men, with a mean age of 33.13 years (SD 16.57). In eight patients we found a normal aerodynamic pattern (in our laboratory; these values range between 70 and 90 mmH_2_O). In six patients the aerodynamic pattern was considered hypofunctional (values < 70 mmH_2_O). In 14 patients the aerodynamic pattern was considered hyperfunctional. The mean value for the sgP was 92.94 mmH_2_O (SD 28.93) The mean for cycle 10 offset was −1.36 ST; the mean for cycle 1 onset was 1.30 ST. We analyzed whether there were statistically significant differences between the RFF of patients with a hyperfunctional pattern and that of patients with a hypo- and normofunctional pattern by means of a one-factor ANOVA. No statistically significant differences were found for any of the three subgroups.

The VFP group included 27 patients: 19 females (70.37%) and 8 men, with a mean age of 56.81 years (SD 15.33). In seven patients we found a normal aerodynamic pattern. In six patients the aerodynamic pattern was considered hypofunctional; in 14 patients the aerodynamic pattern was considered hyperfunctional. The mean value for the sgP was 90.54 mmH_2_O (SD 37.13) The mean for cycle 10 offset was −0.55 ST; the mean for cycle onset was 0.04 ST. No statistically significant differences were found for any of the three subgroups between the RFF of patients with a hyperfunctional pattern and that of patients with a hypo- and normofunctional pattern.

**Figure 2:**
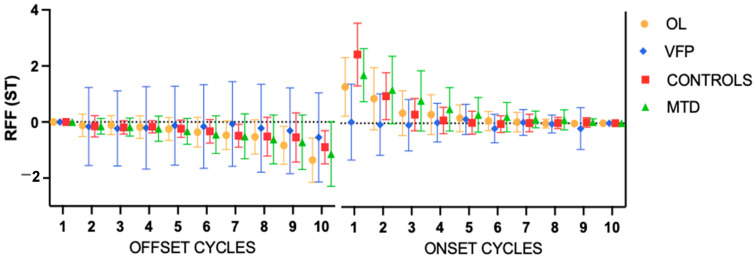
Mean values of the relative fundamental frequency (RFF) for each group. Error bars show the standard error. ST = semitones; OL: organic lesions; VFP: vocal fold paralysis; MTD: muscle tension dysphonia.

Finally, the control group consisted of 15 females (68.19%) and 7 men, with a mean age of 36.19 years (SD 14.57). The mean for cycle 10 offset was −0.9 ST; the mean for cycle onset was 2.46 ST. Figure 2 illustrates the RFF in the ST for each group and Table 2 displays the mean values of each cycle for each group.

### 3.1. Offset Cycles

Two-way ANOVA for offset cycles (Table 3) found statistically significant effects for the groups (*p* = 0.0090) and cycles (*p* < 0.001), and not for the interaction. A Tukey’s test only found significance in the comparison between the VFP and the MTD group and the VFP vs. OL. A Fisher’s test was used to compare different cycles within each group and found statistically significant differences in cycle 10 offset between the OL and the VFP groups (*p* = 0.0006) and between VFP and MTD groups (*p* = 0.0162).

Two-way ANOVA for offset cycles did not find statistically significant differences between the control and non-healthy groups.

### 3.2. Onset Cycles

The two-way ANOVA for onset cycles found statistically significant effects for the groups (*p* < 0.001), cycles (*p* < 0.001), and the interaction (*p* < 0.001). The Tukey’s test found statistically significant differences in all comparisons except the controls vs. the OL group and the controls vs. the MTD group. However, the Fisher’s test revealed statistically significant differences in cycle 1 onset between all groups. Table 4 presents the results of this test for the first cycle.

**Figure 3:**
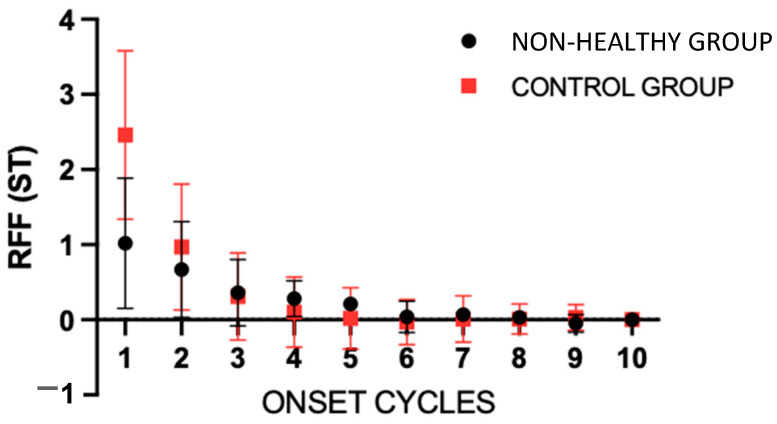
Mean values of the onset relative fundamental frequency (RFF) for the control group and the rest of the groups. Error bars show the standard error. The “non-healthy group” involves the OL, VFP and MTD groups. ST = semitones.

Comparing the control group with the rest of the non-healthy groups (Figure 3), in the two-way ANOVA for onset cycles, statistically significant effects were found for the groups (*p* < 0.001), cycles (*p* < 0.001), and the interaction (*p* < 0.001). Using the Šídák’s multiple comparisons test, statistically significant differences (<0.0001) were found between cycles 1 and 2 of both groups, with a difference of −1.440 ST (−1.727 to −1.153) for cycle 1.

## 4. Discussion

### 4.1. Healthy Voices

As previously mentioned, the hypothesis that RFF could serve as a marker for certain laryngeal pathologies was initially based on changes in $f_{0}$ related to laryngeal adjustments (less laryngeal and hyoid descent) during the onset of vocalization following an unvoiced consonant, which could influence the vocal fold tension [5]. Subsequently, a more in-depth investigation was conducted on the specific changes in each cycle in $f_{0}$ to describe offset and onset phonatory behaviors in similar phonetic contexts, in VCV sequences.

To cease phonation during these vocalic intervals, two primary laryngeal adjustments occurred: vocal fold abduction and an increase in laryngeal muscle tension [14].

Abduction of the vocal folds is primarily attributed to the posterior cricoarytenoid muscle, and reduces transglottic pressure and the duration of the vocal fold contact [15]. Watson subsequently proposed that vocal fold tension increases immediately before the production of voiceless consonants, contributing to the rise in $f_{0}$ at the end of the preceding vowel and the cessation of vibration [16]. Furthermore, he hypothesized that speakers’ tendency to initiate vocal fold abduction before the end of vowel production would lead to a decrease in RFF before the production of the voiceless consonant. The interaction of these two mechanisms could result in a relatively constant fundamental frequency both before and after the production of the voiceless consonant, as he observed in young speakers, and could account for a lower RFF in older individuals, where vocal fold tension is reduced. Therefore, this hypothesis postulates that changes in RFF can be attributed to both laryngeal muscle tension and aerodynamic mechanisms [14].

Stepp et al., 2011 [17], integrated the information from these previous studies to propose a theoretical model for interpreting RFF in speakers with and without voice disorders. The authors hypothesized that the RFF values of offset and onset cycles of vocalization in VCV sequences depend on three laryngeal factors: aerodynamics, muscle tension (as proposed by Watson), and kinematics. The offset cycles of vocalization should generally stabilize around 0 ST in speakers with normal voices, indicating a balance between the increase in laryngeal muscle tension required to cease vocal fold vibration and the reduction in transglottic pressure due to vocal fold abduction for voiceless consonant production.

It has been hypothesized that the RFF values in the cycles farthest from the $f_{0ref}$ and closest to the voiceless consonant, the offset cycle 10 and onset cycle 1, are the most sensitive to aerodynamic, tensional and kinematic factors [18,19,20].

In a scoping review conducted on RFF, they concluded that out of the 14 studies performed with healthy voices to date (2022), offset cycle 10 values ranged from −1.10 to 0.05 ST with a median value of −0.6 ST [21]. The mean of cycle 10 of the control group in our sample is −0.9 ST (SD 0.59), which is consistent with what has been found in the literature to date.

Intervocalic onset behavior is influenced by the preceding voiceless consonant [22]. This is because the laryngeal tension established during the voiceless consonant is transferred to the initial vowel cycles. Existing tension in the cricothyroid and thyroarytenoid muscle is believed to increase $f_{0}$ [23], while vocal fold adduction increases transglottic pressure and helps to reinitiate vocalization.

After several studies on $f_{0}$ changes in VCV sequences [16,24,25], the same phenomenon was observed: $f_{0}$ was generally characterized by a descending pattern at the onset of vocalization, being higher in the first cycles than in the final cycles of the first VCV vowel.

These elevated RFF values observed during the onset cycles are thought to be due to increased transglottal pressure and peak flow during adduction [26] along with elevated longitudinal tension entrained from consonant production. Ladefoged [27] hypothesized that the high rate of airflow at the release of the voiceless consonant may create a large Bernoulli force causing rapid adduction of the vocal folds and thus a higher RFF at the onset. Therefore, the RFF of the vocalization onset cycles in speakers with normal voices is systematically more positive than the values of the final cycles at the onset of the second vowel.

Heller et al. [19] reported a mean of 1.68 ST for onset cycle 1 in healthy voices, which was also the highest value when compared with patients with VH with and without nodules. Roy et al. [28] found values between 2 and 3 ST for onset cycle 1 in controls from their sample, which they compared with patients with MTD. In the scoping review mentioned previously, they concluded that onset cycle 1 values ranged from 1.68 to 3.82 ST, with a median value of 2.60 ST [21].

In our sample, the RFF value for onset cycle 1 in the control group, those without vocal pathology, showed a mean of 2.46 ST, which was the highest among the four groups, thus aligning with values reported in the literature.

### 4.2. MTD Group

In the same scoping review [21], the results obtained for offset cycle 10 in patients with vocal disorders were lower than those in healthy individuals. The RFF has been studied in patients with spasmodic dysphonia [29], Parkinson’s disease [18], and VH with and without phonotraumatic lesions [2,17,28].

MTD, which is attributed to excess laryngeal tension or muscle activation, potentially interferes to a measurable degree with normal RFF patterns because basal levels of laryngeal tension may increase to a point where typical short-term variations in voicing and devoicing patterns are no longer possible [2]. Stepp et al. [17] hypothesized that elevated tension of the laryngeal musculature at the baseline would prevent increased tension levels during the final cycles of vocalization, which would normally help counteract the effects of abduction. It was speculated that the lack of increased tension from increased baseline tension would result in more negative RFF values in the final vocalization cycles compared to adults with normal voices, where values are around 0 ST. In patients with VH -including both phonotraumatic lesions and non-phonotraumatic subgroups, offset cycle 10 ranged from −1.76 ST to −0.80 ST, with a median value of −1.35 ST [21].

In our sample, the mean value of offset cycle 10 for the MTD group was −1.14 ST (SD 1.15), which is also within the range found in the literature, although we did not find statistically significant differences compared to healthy voices. Stepp et al., 2011 [17], obtained a value of −0.80 for offset 10 in patients with VH before vocal therapy, which is also consistent with our results.

Stepp et al., 2010 [2], also hypothesized that the presence of VH would limit the relative increase in laryngeal muscle tension that typically occurs in the vowel following a voiceless consonant, which would also cause a decrease in RFF onset compared with controls. Studying the mechanics of fundamental frequency variation during the phonation onset, Stepp et al. [30] conclude that muscle activation is necessary to produce the observed decay in fundamental frequency evident in vowels preceded by voiceless consonants, and that this is due, in part, to a concomitant decrease in cricothyroid muscle activation during the onset. Competing mechanisms of muscle activation and collision may result in a frequency pattern that initially increases and then decreases. Differences in relative fundamental frequency between healthy and hyperfunctional voices during the onset of vowels preceded by voiceless consonants are derived from the magnitude of the reduction in cricothyroid muscle activation. In addition, they suggest that increased thyroarytenoid muscle activation mitigates the drop in relative fundamental frequency caused by this decrease in cricothyroid muscle activation, which may also contribute to the experimentally observed differences between hyperfunctional and normal phonation.

The onset values in the literature in patients with vocal hyperfunction are in a range between 1.18 and 2.54 ST, with a median of 1.90 ST [21]. As can be seen, there is an overlap with the range of values reported by those with normal voices, which could indicate a poor ability to discriminate this cycle between people with and without HV.

In our study, a median value for onset 1 of 1.72 ST (SD 0.95) was obtained in the MTD group and significant differences were found between all groups in that cycle.

In the studies already mentioned, Stepp et al., 2010 [2], obtained mean values for onset cycle 1 in patients with HV between 2 and 2.5 ST, and in another study in which they compared the results before and after treatment, the mean value obtained was 1.90 ST, in patients with MTD and nodules [17].

However, in contrast to previous observations and our results, Roy et al. [28] found lower RFF values in offset cycles in controls than in patients with MTD, although they did not find significant group effects for offset values.

There are previous reports suggesting that onset RFF may be more sensitive to differences in vocal effort levels than offset values [29]. What we found in this work is that there are group and cycle effects, but no significant differences were found in cycle 10 between most groups. However, our results are aligned with those found in most studies on RFF (with lower offset values for patients with VH). One factor that may have influenced this lack of statistical significance was the number of repeats of the VCV sequences measured. In this study, three repeats were measured for each patient, although there are studies that recommend a minimum of six repeats to obtain greater sensitivity of this parameter [29].

Another factor to consider is the phonetic context of the VCV utterances from which the RFF is extracted. First, the type of stimulus used: a sequence within a speech or an isolated VCV utterance, with uniform repetitions. Second, the RFF can also be affected by the specific voiceless consonant and the vowel. Several different phonemes have been used (e.g.,/f/,/s/,/k/), which may question comparisons across studies. Lien et al. [31] investigated the possible effects of phonetic context on the RFF with the aim of developing a standardized stimulus set for future studies and clinical practice. In typical voices, stimuli containing the voiceless consonants/f/and/ʃ/were found to exhibit the lowest RFF standard deviations and were therefore recommended for RFF analysis.

It should be noted, however, that these phonemes have been studied only within the context of the English language and have never been studied in Spanish. In this study, we used the sequence/pateki/, which is not a Spanish word. However, it has been used in the voice lab of the University of Navarra for years because it is a sequence that presents two VCV utterances (/ate/and/eki/), which, as previously explained, are characteristic patterns during phonation.

### 4.3. OL Group

Stepp et al. 2010 [2] investigated RFF in patients with VH, comparing four groups: patients with polyps, with nodules, with MTD, and controls. They found significant differences between the groups, with an offset cycle 10 around −1 ST for the MTD group. Patients in the polyps and nodules groups had even lower offset 10 values, around −1.5 ST. Heller Murray et al. [19] distinguished the effects of longitudinal and transverse laryngeal muscle tension in specific patient populations. They proposed that patients with phonotraumatic injuries may depend on elevated longitudinal and transverse tension that interacts with the laryngeal kinematics proposed in the model of Stepp et al., 2011 [17]. The additional transverse tension—due to injury—of the vocal folds would increase the contact time between them, thus reducing the effects of vocal fold abduction and consequently decreasing the RFF values beyond the effects of the longitudinal tension alone. Similarly, it was thought that increased transverse tension would reduce the duration of the adductor gesture and inhibit the impact of aerodynamic forces on the RFF to, again, reduce RFF values compared with patients with only elevated longitudinal tension at the baseline.

Heller Murray et al. [19] found a mean value of 1.18 ST in patients with HV with phonotraumatic lesions and 1.59 ST in patients with HV without lesions, with significant differences between these groups and the controls. In addition, Roy et al. confirmed that the slopes of RFF onset varied systematically as a function of group membership (pMTD vs. controls), supporting the RFF onset as an objective acoustic measure of vocal hyperfunction in a subset of disordered voices [28].

In our sample, the mean value of offset cycle 10 for patients with OL was −1.36 ST (SD 0.79) and the mean value of onset cycle 1 was 1.30 ST (SD 1.05). As can be seen, patients with polyps/nodules present lower values than patients with MTD, in agreement with the above-mentioned theory.

Traditionally, dysphonia due to organic lesions, whether polyps or nodules, is usually associated with hyperfunctional aerodynamic patterns. However, this association does not necessarily occur. We can find any type of aerodynamic pattern in these patients, and so we found it in our sample. Although most patients have elevated subglottic pressure, we also found others with normal or even low pressures. This may be influenced by different factors. One issue to consider is that the origin is not always the same. We often refer to secondary MTD in patients with lesions in the mucosa of the vocal folds, since these impede the proper closure of the folds, which would require a hyperfunction of the muscles involved in phonation to try to compensate for this lack of closure. However, the mechanism could also occur in reverse: with dysphonia due to muscle tension, excessive force during phonation could lead to traumatic injury to the vocal fold mucosa. In the first case, we might find normal or even low subglottic pressure before the patient compensates for the vocal fold closure deficit.

Most of the studies conducted thus far on RFF involve patients with hyperfunctional pathology. There are also studies indicating that this type of voice is associated with elevated subglottic pressures during phonation [32,33,34,35], which can only be measured by performing an aerodynamic analysis.

If, as demonstrated, patients with vocal hyperfunction exhibit lower RFF values than healthy individuals, it could be expected that we would find an association between different aerodynamic patterns (normal, hypo-, and hyperfunctional) and the RFF.

However, this association was not found in our sample. In the case of patients with OL, the theory of Heller Murray et al. [19] mentioned previously can justify the fact that in these patients, the RFF may depend on the longitudinal and transverse strain and abduction more than the aerodynamic pattern. Additionally in these patients, the Bernoulli effect, caused by the high airflow velocity at the release of the voiceless consonant, decreases as the mass of the vibratory element increases.

### 4.4. VFP Group

An interesting finding not discussed yet is the RFF values of the VFP group. To our knowledge, there are no studies to date that have studied this parameter in these patients. Among the four groups studied, this is the one with values closest to 0 ST in both offset and onset.

VFP causes fundamental frequency irregularities, weak respiratory dysphonia, hoarseness, and limitations in speech loudness [36]. Unilateral VFP decreases the vocal fold tension, often resulting in dysphonia and an aspirated voice [37], indicating the need for an exaggerated aerodynamic compensatory mechanism to produce an adequate acoustic output [38]. However, in the case of vocal fold paralysis we can also find different aerodynamic patterns. It would be logical to think that a patient with paralysis of a vocal fold would have a decreased subglottic pressure (hypofunctional pattern), due to incomplete closure of the folds during phonation, and the consequent air leakage. Nevertheless, we know that with time these patients attempt to compensate the paralysis with the contralateral intrinsic and extrinsic musculature and part of the homolateral musculature, and eventually they present complete closure, or a supraglottic closure. This could result in normal or even elevated subglottic pressures.

For offset, the levels around 0 ST could be justified because there is still a balance between laryngeal tension and transglottic pressure, both of which are almost nonexistent. In this case, there is no tension in the vocal fold due to the lack of innervation of the thyroarytenoid muscle, and the decrease in pressure is less pronounced because, although the transglottic flow increases, the basal pressure—before devoicing—is lower, due to the missing tension and/or reduced contact between both vocal folds. For the onset, values also around 0 ST could be explained in a similar way. There was no significant increase in either laryngeal tension or transglottic pressure, as in typical voices. Therefore, there was no effect on the RFF. It is also worth mentioning that, in these patients, there is an effect that is not usually mentioned, and that is the fact that paralysis not only generates an asymmetry in terms of the horizontal movement of the vocal folds, but can also lead to an asymmetry in terms of the height of one vocal fold and the other [39]. In this sense, aerodynamic forces do not occur in the same way as in healthy vocal folds. The Bernoulli effect that we have mentioned, which takes place after the voiceless consonant, will be altered and may, for this reason, have less influence on the RFF in these patients.

However, the laryngeal biomechanics and consequent aerodynamic pattern may vary depending on the level at which the nerve injury has been produced. For example, it will not be the same if in addition to a recurrent lesion there is damage to the external branch of the superior laryngeal nerve, since this will also affect the cricothyroid muscle and, therefore, the longitudinal tension of the vocal cord. It could therefore be interesting in the future to study the variations in this parameter in a sample of patients with vocal cord paralysis characterized according to the type of nerve injury.

### 4.5. Limitations

One of the limitations of this study is that only three repeats of/pateki/were used for each patient, although the minimum suggested in the literature is six [29]. This is because the recording protocol that has been used for decades in the voice laboratory of the University of Navarra only included three repeats of this phoneme. Three more have now been added to the protocol. For the same reason, only this sequence—/pateki/—has been analyzed to study the VCV context, not having others to compare the results with. This also results in another limitation since it is a phoneme that has not been previously studied. Therefore, comparisons with the results found in the literature must be made cautiously.

Finally, another limitation for RFF extraction is that there may be cases where it cannot be assessed, especially when the voice quality is particularly poor.

## 5. Conclusions

It can be concluded that the RFF is an indicator that provides relevant information about the biomechanics of the phonatory pattern and seems to be able to differentiate between healthy and diseased voices, and even between different voice disorders. Our results suggest that the RFF in onset cycles is more suitable for this purpose. It may be an affordable and accurate indicator that allows us to objectively identify biomechanical disorders without resorting to complex aerodynamic analysis techniques.

The RFF patterns had been previously studied for different voice disorders, but they have not been previously described in patients with VFP, where values around 0 ST in both offset and onset cycles are found. These findings suggest that this parameter extracted from acoustic analysis, the RFF, may be of interest in pathologies that are not necessarily related to vocal hyperfunction.

As the study was conducted retrospectively, with the limitations that this entails, this work is considered a pilot study that reinforces the interest that this parameter (RFF) may have in the diagnosis and monitoring of different voice disorders, and opens the door to future lines of research with more extensive studies and more complex methodologies that will strengthen its clinical value, such as studying the most appropriate phonemes in the context of languages other than English, in normal and pathological voices or its variation in other pathologies with alterations in laryngeal biomechanics not necessarily related to vocal hyperfunction.

Future research is needed in this area to further develop its clinical application, such as studying the most appropriate phonemes in the context of languages other than English, in normal and pathological voices, its variation in other pathologies with alterations in laryngeal biomechanics not necessarily related to vocal hyperfunction, or its value for monitoring the treatment of different voice disorders.

## Figures and Tables

**Table 1:** RFF offset calculation example.

Glottal Pulse	Glottal Pulse Time (sec)	Period (sec)	f_0_	RFF (ST)
**1**	4.903451915	0.007246401987	138.00	0.00
**2**	4.910698317	0.007242313392	138.08	0.01
**3**	4.917940631	0.007248108619	137.97	0.00
**4**	4.925188739	0.007251200303	137.91	−0.01
**5**	4.93243994	0.007260750708	137.73	−0.03
**6**	4.93970069	0.007271711372	137.52	−0.06
**7**	4.946972402	0.007282732213	137.31	−0.09
**8**	4.954255134	0.007220881634	138.49	0.06
**9**	4.961476016	0.007630824667	131.05	−0.89
**10**	4.96910684	0.007432050926	134.55	−0.44
**11**	4.976538891			

**Table 2:** RFF (ST) for offset and onset cycles.

CYCLE		MTD		OL		VFP		CONTROLS	
		$\bar{x}$	SD	$\bar{x}$	SD	$\bar{x}$	SD	$\bar{x}$	SD
**offset cycles**	1	0	0	0	0	0	0	0	0
	2	−0.15	0.28	−0.12	0.4	−0.16	1.39	−0.15	0.38
	3	−0.18	0.32	−0.11	0.34	−0.23	1.34	−0.19	0.24
	4	−0.24	0.45	−0.18	0.4	−0.21	1.47	−0.17	0.22
	5	−0.33	0.46	−0.25	0.41	−0.13	1.41	−0.24	0.31
	6	−0.45	0.67	−0.36	0.53	−0.16	1.49	−0.33	0.42
	7	−0.51	0.8	−0.47	0.51	−0.07	1.51	−0.49	0.41
	8	−0.62	0.87	−0.53	0.61	−0.22	1.57	−0.52	0.69
	9	−0.72	0.97	−0.83	0.68	−0.31	1.53	−0.55	0.88
	10	−1.14	1.15	−1.36	0.79	−0.55	1.59	−0.9	0.59
**onset cycles**	1	1.72	0.95	1.3	1.05	0.04	1.36	2.46	1.12
	2	1.19	1.21	0.88	1.11	−0.05	1.1	0.97	0.84
	3	0.8	1.08	0.36	0.8	−0.07	0.92	0.31	0.58
	4	0.5	0.78	0.31	0.71	0.02	0.69	0.1	0.47
	5	0.3	0.62	0.18	0.48	0.14	0.54	0.02	0.41
	6	0.22	0.53	0.08	0.34	−0.19	0.51	−0.03	0.3
	7	0.14	0.3	0.04	0.35	0.03	0.46	0.01	0.31
	8	0.12	0.36	−0.01	0.16	−0.03	0.32	0.01	0.2
	9	0.04	0.12	−0.01	0.1	−0.19	0.75	0.03	0.17
	10	0	0	0	0	0	0	0	0

MTD: Muscle Tension Dysphonia; OL: Organic lesions; VFP: Vocal fold paralysis; $\bar{x}$: average; SD: standard deviation.

**Table 3:** Results of two-way analysis of variance on offset relative fundamental frequency values.

EFFECT	SS (Type III)	DF	MS	F	*p* Value
Vocal cycle x group	12.91	27	0.4781	0.6275	0.9308
Vocal cycle	71.69	9	7.966	10.46	<0.0001
Group	8.862	3	2.954	3.877	0.0090

SS: sum of squares; DF: degrees of freedom; MS: mean squares.

**Table 4:** Fisher’s LSD for onset cycle 1.

	Predicted (LS) Mean Diff.	95.00% CI of Diff.	Individual *p* Value
Cycle 1			
OL vs. VFP	1.26	0.9050 to 1.615	<0.0001
OL vs. CONTROLS	−1.16	−1.535 to −0.7850	<0.0001
OL vs. MTD	−0.42	−0.7861 to −0.05389	0.0246
VFP vs. CONTROLS	−2.42	−2.798 to −2.042	<0.0001
VFP vs. MTD	−1.68	−2.049 to −1.311	<0.0001
CONTROLS vs. MTD	0.74	0.3515 to 1.128	0.0002

## Data Availability

All data generated or analyzed during this study are included in this article. Further inquiries can be directed to the corresponding author.
